# Cell and tissue manipulation with ultrashort infrared laser pulses in light-sheet microscopy

**DOI:** 10.1038/s41598-019-54349-x

**Published:** 2020-02-06

**Authors:** Gustavo de Medeiros, Dimitri Kromm, Balint Balazs, Nils Norlin, Stefan Günther, Emiliano Izquierdo, Paolo Ronchi, Shinya Komoto, Uros Krzic, Yannick Schwab, Francesca Peri, Stefano de Renzis, Maria Leptin, Matteo Rauzi, Lars Hufnagel

**Affiliations:** 1 European Molecular Biology Laboratory Heidelberg, Meyerhofstrasse 1, 69117 Heidelberg, Germany; 2 Université Côte d’Azur, CNRS, Inserm, iBV, Nice, France; 3 Collaboration for joint PhD degree between EMBL and Heidelberg University, Faculty of Biosciences, Heidelberg, Germany; 4 Present Address: Luxendo GmbH, Kurfürsten-Anlage 58, 69115 Heidelberg, Germany; 5 Present Address: Institute of Molecular Life Sciences, University of Zurich, Winterthurerstrasse 190, CH-8057 Zurich, Switzerland; 6 Present Address: Friedrich Miescher Institute for Biomedical Research, Maulbeerstr. 66, CH-4058 Basel, Switzerland; 7 Present Address: Imaging Section, Okinawa Institute of Science and Technology, 1919-1 Tancha, Onna, Okinawa 904-0495 Japan

**Keywords:** Laser material processing, Light-sheet microscopy

## Abstract

Three-dimensional live imaging has become an indispensable technique in the fields of cell, developmental and neural biology. Precise spatio-temporal manipulation of biological entities is often required for a deeper functional understanding of the underlying biological process. Here we present a home-built integrated framework and optical design that combines three-dimensional light-sheet imaging over time with precise spatio-temporal optical manipulations induced by short infrared laser pulses. We demonstrate their potential for sub-cellular ablation of neurons and nuclei, tissue cauterization and optogenetics by using the *Drosophila melanogaster* and zebrafish model systems.

## Introduction

Imaging the development of entire living organisms at high spatial resolution over long periods of time allows a descriptive analysis of biological processes taking place from within the cell to the full animal scale. Light-sheet fluorescence microscopy has proven to be a powerful imaging technique for live 3D imaging due to its gentle illumination scheme, high spatial resolution and fast data acquisition^1–5^. However, observation alone is not always sufficient to directly probe biological processes. Tools to manipulate, control and perturb biological processes are necessary to gain an understanding of the underlying mechanisms.

In the wide spectrum of tools available for chemical, genetic and physical manipulations, laser-based manipulation stands out for its high spatio-temporal specificity and biological selectivity^6^. Laser-based manipulation indicates the ability to perturb living matter using laser. Applications of optical manipulation techniques in biology can broadly be classified in (i) interactions with molecular specificity and (ii) non-molecule specific interactions. Only the first group relies on the expression of specific optogenetic constructs^7^. Application of these techniques include optical control of neuronal activities^8^, modulation of cytoskeletal components^9–13^, probing cell and tissue mechanical properties^14^, DNA damage^15^, cell apoptosis^15^, dissection of cell-cell contacts^16^ and perturbation of tissue interactions^17^. Laser manipulation typically relies either on pulsed or continuous laser sources in the ultra-violet (UV) or infrared (IR) range.

Early work showed that by tightly focusing a high power laser it is possible to selectively ablate structures in a living embryo^17^ or even within cells, for instance mitochondria^18^, microtubule filaments^19^ or actin stress fibers^11^. Several studies used laser-based manipulation to study tissue morphogenesis and cell mechanics in developing embryos: for instance this technique was the first to allow the precise ablation of actomyosin networks at cell-cell junctions within an epithelium^16,20^. Laser-based manipulation is also a useful tool to dissect single axons in developed animals for behavioral studies as demonstrated for instance in early work on laser axotomy in *C. elegans*^21,22^. This technique was also used to perturb morphogenetic movements in the developing *Drosophila* embryo^23^. With the advent of optogenetics^24^, this technique has been growing in popularity and is now used extensively to perturb living samples with spatio-temporal and protein specificity.

The potential of a laser-based manipulation system does not rely only on the laser manipulation module. Instead, the imaging system to which the laser module is coupled plays a key role. Confocal microscopes allow, for instance, to monitor fluorescent structures after laser manipulation. Fast confocal systems (based on spinning disc technology) allow to record, with sub-second time resolution, rapid cytoskeleton recoil after local laser ablation. Finally, light-sheet microscopy allows to capture fast and long-lasting 3D time-lapses of a cell^25^ or of an entire embryo^26^ before and after the laser manipulation.

IR wavelengths provide several advantages for laser manipulation: 1) IR photons are less sensitive to refractive index mismatch compared to lower wavelengths. This allows targeting any region of an embryo with no need of refocusing the beam; 2) because of the low energy of IR photons, IR femtosecond pulses generate non-linear absorption exclusively in the focal volume of the objective where the laser intensity is the highest: this allows laser manipulation in a 3D confined space; 3) multi-photon processes can be induced such as plasma induced ablation^27^, laser cauterization^26,28^ and two-photon optogenetics^12,13^: these processes can be applied in different biological contexts to probe the properties of the living matter from the subcellular to the embryo scale.

Here we describe a home-built integrated framework and an optical design that combines IR femtosecond laser based photo-manipulation with multiview light-sheet imaging (MuVi-SPIM^2^). Our set-up allows to target any region within an embryo while simultaneously visualizing the sample in 3D with high temporal resolution (Supplemetary Note 3). We demonstrate the performance of the system in various applications of laser dissection, laser cauterization and optogenetics in *Drosophila melanogaster* and zebrafish embryos.

## Results

### Optical set-up

**Figure 1:**
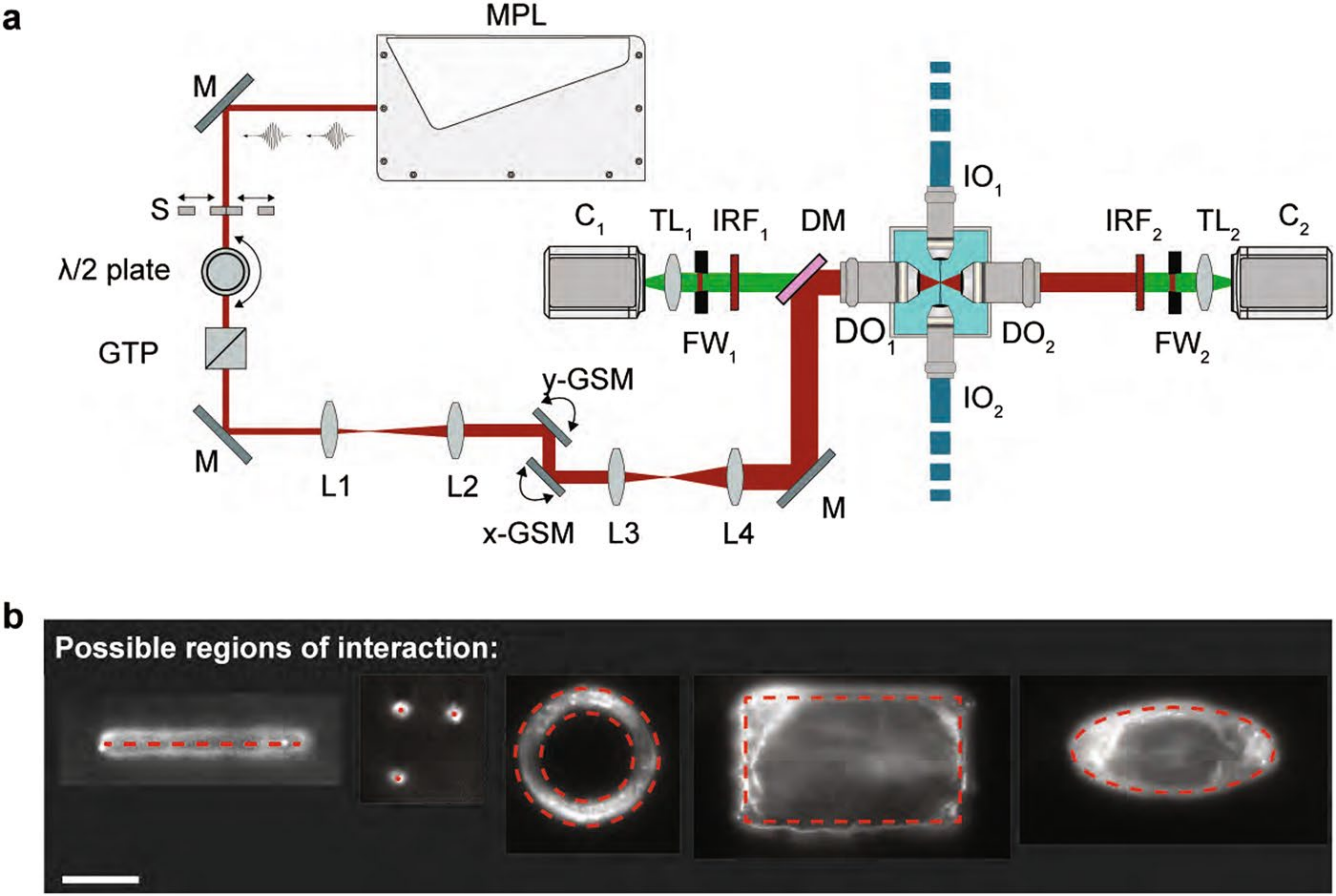
Scheme of the optical set-up and examples of possible ablation regions. (**a**) The multiphoton ablation unit is shown on the left part of the sketch, part of the the multiview light-sheet microscope is shown on the right. The multiphoton laser (MPL) produces ultrashort pulses that can be blocked or transmitted with the help of a shutter (S). A motorized λ/2 plate in combination with a Glan-Thompson prism (GTP) is used to control the power of the laser. Following the power control unit, the beam is expanded 2-fold via a beam expander (L1, L2).The galvanometric scan mirrors (y-GSM, x-GSM) are located near the conjugated plane of the detection objective’s back aperture. Therefore, their rotation corresponds to a rotation in the back aperture of the detection objective. Next, the beam is expanded 8-fold by a second beam expander (L3, L4) to fill the entire back aperture of the detection objective. A dichroic mirror (DM) finally redirects the infrared light towards the back aperture of the detection objective (DO_1_) while still allowing fluorescent signal to be detected. Several mirrors (M, not all illustrated) are used to redirect the infrared laser towards the dichroic mirror. The multiview light-sheet microscope has two illumination objectives (IO_1_ and IO_2_) and two detection objectives (DO_1_ and DO_2_). Each detection arm additionally contains a filter wheel (FW_i_), tube lens (TL_i_), and a camera (C_i_), i = 1, 2. To prevent possible infrared light to reach the cameras, two infrared filters (IRF) are added to each detection arm. **(b)** The custom-made software allows to define 5 different types of region of interest (ROIs), which can be drawn by the user: linear, punctual, elliptical/circular, coronal or rectangular. Red dashed lines indicate the ROIs used for the resulting ablations performed on a nylon fiber and imaged with 488 nm excitation laser. Scale bar is 25 μm.

IR light can penetrate deeply into tissues since it suffers less from scattering and aberrations than shorter wavelengths^29,30^. To induce multi-photon absorption, high photon densities are necessary. The latter can be achieved by focusing fs laser pulses with high numerical aperture (NA) lenses. For our system we used a 180 fs pulsed IR laser coupled to a 1.1 NA detection objective of our MuVi-SPIM set-up to focus the pulses on the imaging plane. The full optical path is shown in Fig. 1a and a full description of the optical parts and the optical arrangement is discussed in Supplementary Note 1.

The energy delivered by each laser pulse (details in Supplementary Table 1) can be calculated by using the equation *E*_*pulse*_ = *P*_*avg*_ / *f*_*rep*_, where *E*_*pulse*_ is the energy of the pulse, *P*_*avg*_ the average laser power and *f*_*rep*_ the pulse repetition rate. The maximum pulse energy that our laser delivers is 20.3 nJ. 50% of this energy is lost in the optical path (with more than 40% in the objective). Additionally, at maximum average power, the estimated pulse peak power density is of the order of 10^12^ Wcm^−2^. This value shows that our system has the prerequisites to perform photo-ablation on cells or tissues^31^. In all experiments shown below, the measurement of average laser power was performed at the back aperture of the objective. To allow interactive manipulations of the sample, a custom software has been developed (Supplementary Note 3), which allows the user to draw a region of interest (ROI) of different shape (linear, punctual, elliptical/circular, coronal or rectangular) directly on the acquired images (Fig. 1b). The software will then automatically calculate the position of the galvanometric scan mirrors (GSM) over time defining the beam trajectories necessary to scan the ROI selected. Finally, our system presents two main advantages: (i) in comparison to seminal work that implemented laser based manipulation on light-sheet microscopes^25^, we use a IR femtosecond laser that allows to photo-manipulate in deep tissue at the imaging plane for simultaneous recording of sample perturbation. (ii) In contrast to recent work, also using IR femtosecond laser coupled to light sheet microscopy^32^, we use a high NA objective (1,1 NA) resulting in an increased confinement of the laser intensity distribution along the z axis. This implies a more localized photo-manipulation with reduced surrounding damage. For cellular/subcellular manipulation it is crucial to minimize the extent of the ablation in all three dimensions. Lower NA objectives suffer from poor axial resolution (e.g., NA = 0.5 has an axial resolution >4 µm) making a single cell ablation more unlikely. We present here several applications of this tool on two embryonic model systems.

### Ablation of individual nuclei in the early *Drosophila* embryo syncytium

After fertilization, the primordial nucleus, located in the center of the *Drosophila* embryo, undergoes 14 mitotic divisions without cytokinesis, resulting in a syncytium with approximately 6000 nuclei. At mitotic division cycle 10, ~70% of the nuclei reach the cortex of the embryo. The remaining nuclei move back to the central region of the yolk cell^33^.

The nuclei form a highly regular array in the cortical region of the embryo. So far it has not been possible to study the mechanism that determines their spatial distribution. One way of doing this is to interfere with their packing by preventing individual nuclei from reaching the surface in a selective manner. We thus performed targeted ablation of selected nuclei before mitotic division 10 to see whether damaged nuclei can reach the cortex after mitotic division 9. We used our home-built MuVi-SPIM to image embryos expressing an mRFP-tagged nucleoporin (NUP107^34^). NUPs are part of the nuclear pore complex (NPC), which efficiently marks the outline of nuclei. By using IR ablation coupled to MuVi-SPIM, we could photodamage any nucleus at any of the mitotic cycles starting from mitotic cycle 7 and follow their movements at high 4D resolution.

**Figure 2:**
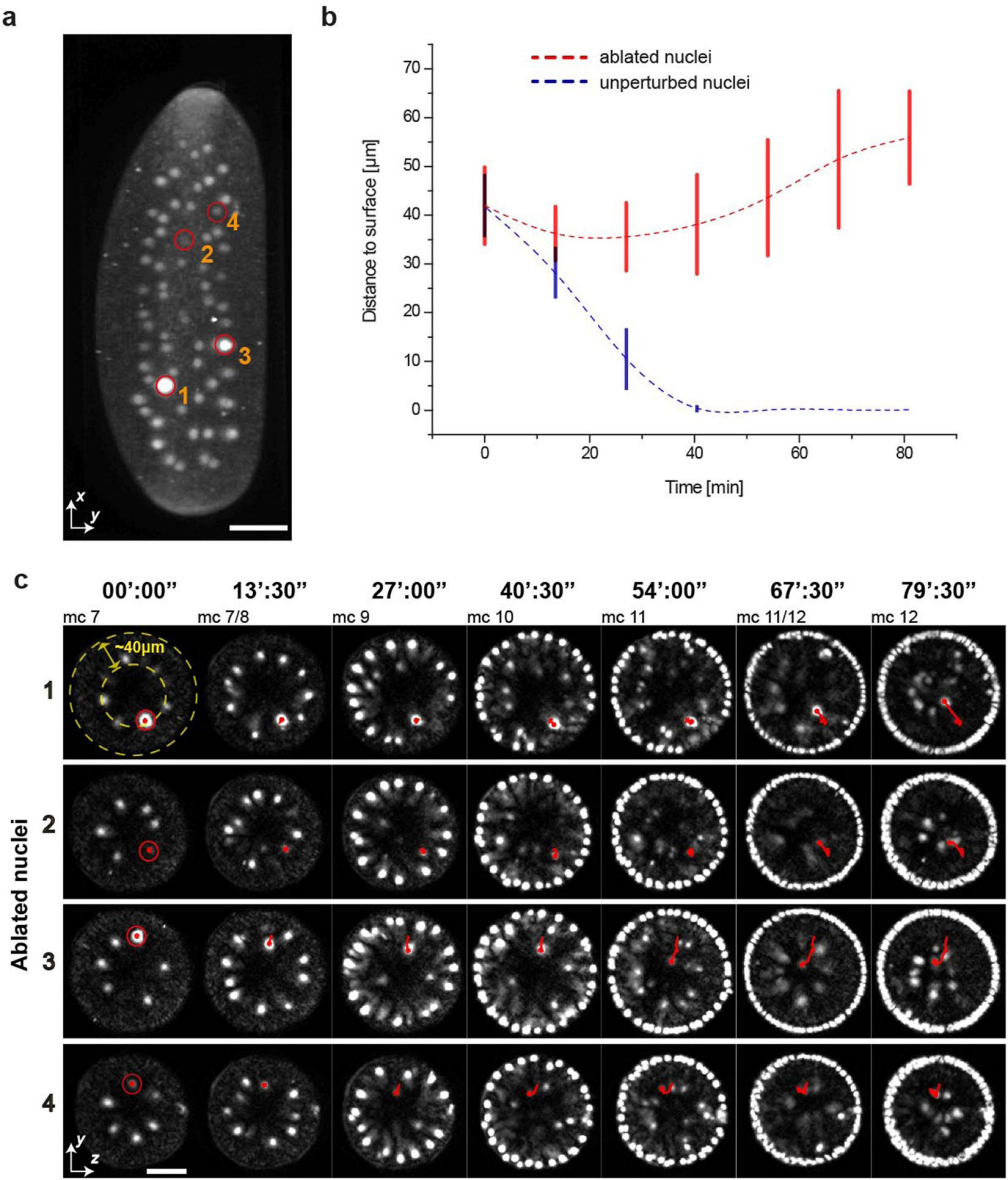
Bulk ablation of nuclei in early *Drosophila* development. (**a**) Projection of a *Drosophila* embryo at mitotic cycle 7 shows all 64 nuclei marked with NUP107-mRFP, with 4 ablated nuclei encircled in red, their numbering being used for the timelapse shown in c. (**b**) 17 ablated nuclei in 5 different embryos failed to reach the surface of the embryo at mc 10 (red line shows average nuclear displacement from the surface and error bars represent the standard deviation), whereas unperturbed neighboring nuclei move to the surface (blue line). Representative mitotic cycles are described as mc#, # being the corresponding cycle number. **(c)** 40 µm thick projections of transversal sections of the 4 ablated nuclei encircled in (**a**), aligned so that ventral is facing down. The nuclei are localized at distance of ~40 µm inside the embryo. Although at similar distance from the posterior side, nuclei 1 and 3 have been selected at almost opposite positions (more ventral to more dorsal, respectively) by rotating the sample. Selected time points depict dynamics of ablated nuclei as they move back to the inside of the embryo (red tracks). Scale bars are 50 μm.

Figure 2 shows a nuclear ablation experiment in a *Drosophila* embryo at mitotic cycle 7 (i.e. at the 128 nuclei stage). The average depth of the nuclei from the egg surface at mitotic cycle 7 was measured to be 40 μm and at the end of mitotic cycle 7 the depth decreased to 27 μm (in agreement with previous studies^35^). We ablated 4 nuclei (Fig. 2a) and imaged the entire embryo for one hour (Fig. 2c). All four ablated nuclei failed to reach the cortex of the embryo and moved back to the center of the yolk cell whereas in most cases the trajectories of neighboring nuclei were not affected (Fig. 2b,c and Supplementary Movie 1). The experiment was repeated 19 times in 5 different embryos, and in 18 cases the ablated nuclei failed to reach the cortex, while the neighboring nuclei moved to the cortex of the embryo. Ablated nuclei persisted in a more central region of the embryo inside the yolk. For all these experiments the imaged embryos transitioned from mitotic cycle 7 to mitotic cycle 8 in less than 10 minutes in agreement with previous studies on fixed samples^35,36^ supporting the idea that the 3D imaging performed with MuVi-SPIM at a rate of 4 stacks per 90 s did not perturb the process analyzed.

In conclusion, IR laser ablation coupled to MuVi-SPIM is an effective tool to selectively perturb and track in 4D nuclei during early embryonic mitotic divisions.

### Localized neuronal ablation and damage response

**Figure 3:**
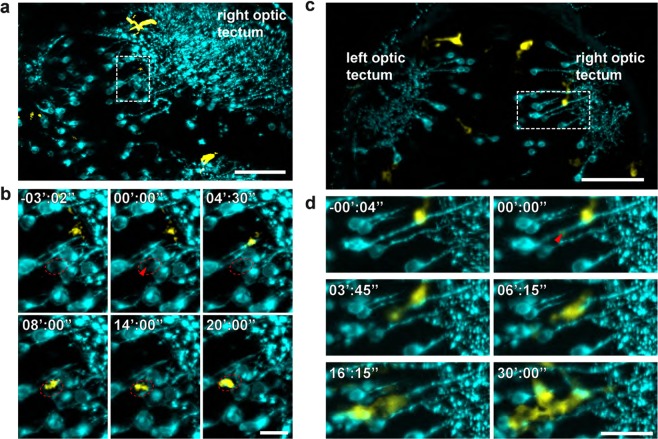
Single soma and axon dissection in the brain of a 5 day post fertilization zebrafish expressing Synaptophysin-GFP. (**a,c**) Maximum intensity projections of the regions encompassing the ablation volumes, depicted in dashed boxes. Scale bars are 50 μm. (**b**) Time lapse of single soma ablation within the dashed box shown in a), where the neighboring neuron remains intact. Localization of the ablation spot is depicted within the dashed red ellipse, by the red arrow. As a result, the Synaptophysin signal is reduced, and a microglia interacts with the ablated soma, reaching the region around 8 minutes after ablation, from ~22 µm distance. 0 seconds corresponds to the time of ablation. Scale bar is 10 μm. **(d)** Time lapse of an axon cut. 6 minutes after laser ablation a microglia, located 15 µm away, moves to the ablation site, shortly followed by two others. Cyan: Synaptophysin-sfGFP, yellow: pu.1-RFP. Scale bar is 20 μm.

We further tested our system on the zebrafish embryo brain to selectively target neurons and monitor the response of microglia. Microglia are brain-resident macrophages that perform clearance tasks for the maintenance of a healthy environment, one of them being phagocytosis of apoptotic neurons^37^. In previous studies injuries of about 100 μm in diameter were made in the fish brain using a UV laser coupled to a confocal microscope to investigate the signals that guide microglia towards brain damage^38^. Recent work on the neuronal function in live zebrafish used an optical setup similar to the one presented here to perturb the brain circuit^32^. We now used the IR pulsed laser to selectively ablate either the somata or the axons of neurons and monitored the response of microglia lying deep inside the brain. We marked the cells with Synaptophysin-sfGFP (see methods section). In this transgenic line, the expression of the fluorescent marker is patchy allowing easy visualization of single neurons (see Fig. 3a,c).

To test the spatial precision of our dissecting system we targeted one single soma located in close proximity to other neurons at 80 µm depth in the brain (see Fig. 3a,b). By using an average power of 880 mW and 5 times 9 ms exposure time we generated a micro-cavitation bubble inside the neuronal cell body. After ablation, the targeted soma lost much of its fluorescent signal and 20 minutes later a microglial cell, originally positioned 23 µm away from the injury, reached the ablated soma (Supplementary Movie 2**)** moving at a speed of 3 μm/min (consistent with previous measurements^38^). The neurons neighboring the ablated soma were not affected since the micro-cavitation bubble was confined inside the ablated soma. This was confirmed by the fact that microglia approached the ablated neuron but not the neighboring ones.

We then dissected an axon, using an average power of 604 mW at the back aperture of the detection objective and 3 ms exposure time. The ablated axon was located at 65 μm depth, near the right optic tectum, as shown in Fig. 3c. The axon retracted after ablation, and at 8.5 min post-dissection the remaining part of the damaged axon was dragged away by one microglia initially positioned 15 μm away from the ablated site. Four microglia reached the damaged axon (Fig. 3d and Supplementary Movie 3) at average speeds similar to those measured during soma ablation. Finally, IR laser ablation coupled to MuVI-SPIM is an effective tool to selectively dissect soma with simultaneous imaging and eventual long lasting 3D time-lapse recording with low photo bleaching and photo toxicity to monitor microglia behavior after soma ablation.

### Laser cauterization: establishing immobile boundaries in tissues

**Figure 4:**
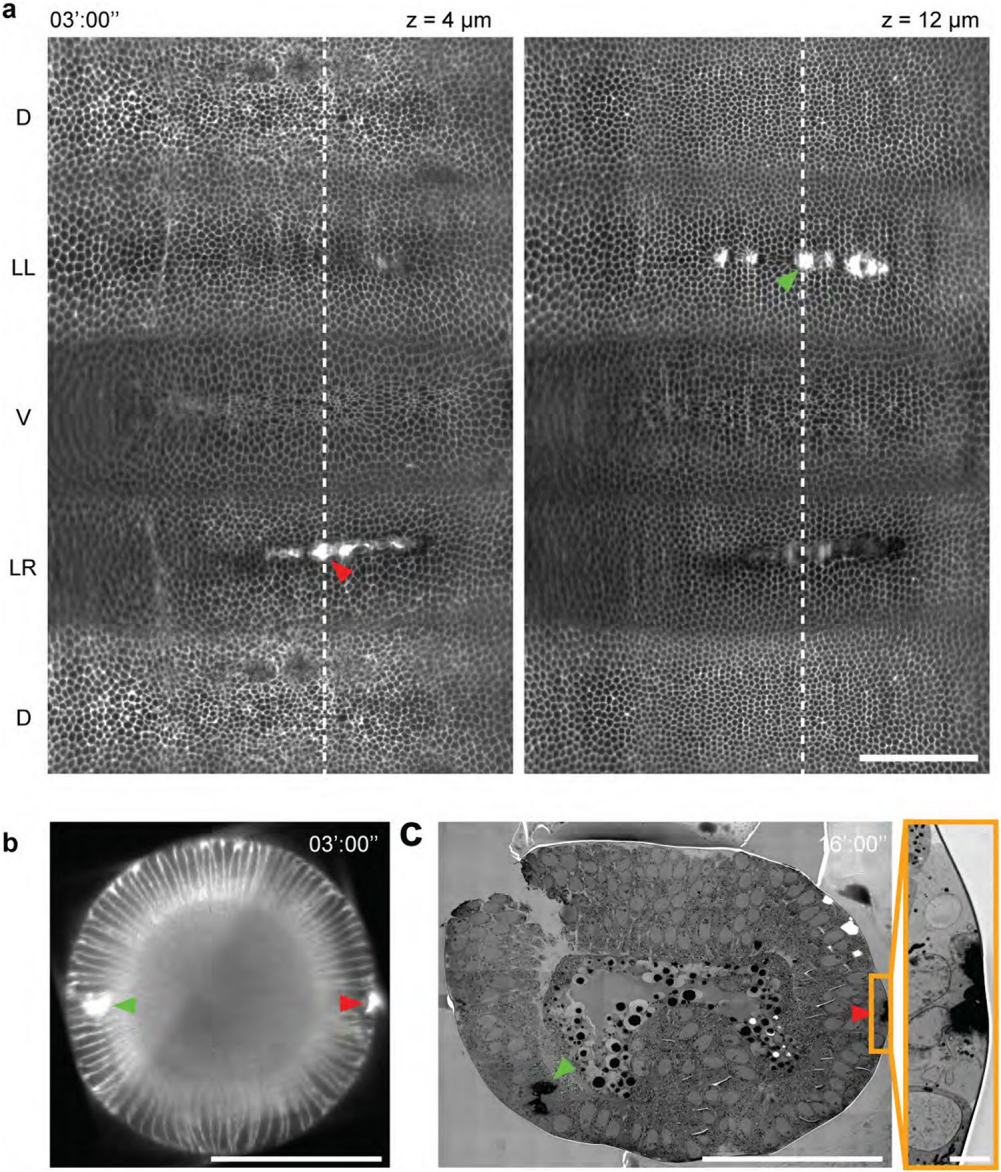
Tissue cauterization. (**a**) 2D cylindrical map projection of the entire embryonic epithelium at 4 µm depth (left panel) and at 12 µm depth (right panel) below the surface of a *Drosophila* embryo on which two cauterizations were performed, marked with yellow and red arrows. The time of cauterization was 3 minutes after ventral furrow onset. (**b**) Cross-section view of the embryo along the dashed line in (**a**). (**c**) Electron-microscopy overview image of the embryo in (**a**) 16 minutes after the onset of gastrulation. The right panel shows a higher magnification of the cauterized region on the right side of the embryo (yellow box in the overview). Scale bar 100 μm in (**a–c**), and 5 μm in insert.

While IR fs laser sources have been extensively used for laser dissection^16,39^, recent work has shown that this laser source, at lower power and longer exposure times, can be used to cauterize tissues and establish immobile boundaries in the *Drosophila* embryo^26,28^. Laser cauterization at the surface of the embryo induces the tethering between targeted cells and the vitelline membrane that surrounds the embryo. Since the vitelline membrane does not move during development and since it is sufficiently rigid, the tethering of cells to the vitelline membrane results in the formation of immobile boundaries that can interfere with cellular flows and tissue morphogenesis. Since IR laser cauterization is confined in the focal volume this can be induced at different depths in the tissue (Fig. 4a–c). Nevertheless, immobile boundaries are induced only when cauterization is performed at the interface between the epithelium and the vitelline membrane (Fig. 4b, right side); cauterization *per se* will not induce the formation of a fixed boundary.

During *Drosophila* gastrulation, the left and right ectodermal regions flow towards the ventral side allowing ventral tissue internalization^26^. In the experiment presented in Fig. 4 cauterization was performed both on the left and right side. While the right side was cauterized at the interface between the epithelium and the vitelline membrane, on the left side this was performed in the underlying cytoplasm at 20 µm from the surface. Both cauterizations result in a local increase in electron dense material seen in transmission electron micrographs (Fig. 4c). In this experiment, the cauterized cells on the right were immobilized, while those on the left were still free to move, which allowed the ventral tissue to internalize (Supplementary Movie 4 and 5) in agreement with previous studies^26^. Finally, IR laser coupled to MuVi-SPIM is an effective tool to induce tissue cauterization and monitor eventual tissue flow perturbation at the embryo scale with cellular resolution. Only if cauterization is performed at the interface between the tissue and the membrane enveloping the embryo, this can generate fix boundaries that can impede tissue flow.

### Localized optogenetic manipulations together with *in toto* imaging

Optogenetics allows the localization or the activity of a protein to be modulated by light^7^. This is achieved by fusing the protein of interest with a photosensitive unit that, under light exposure, can change its conformation inducing heterodimerization^40,41^, homodimerization^42^, oligomerization^43^ or photo-uncaging^44^. With optogenetics it is possible to modulate cell signaling, motility, contractility, apoptosis, differentiation, etc. with spatial and temporal specificity^7,45^.

To demonstrate the use of *in toto* imaging coupled with optogenetics we first used the CRY2-CIB1 system to control the localization of the catalytic domain of the inositol polyphosphate 5-phosphatase OCRL to deplete PI(4,5)P_2_ and thus F-actin from the cell cortex in *Drosophila*^13^ while imaging the entire embryo with MuVi-SPIM and electronic confocal slit detection^46^. To increase the axial resolution of photoactivation, IR fs light can be used to induce two-photon absorption^13^.

**Figure 5:**
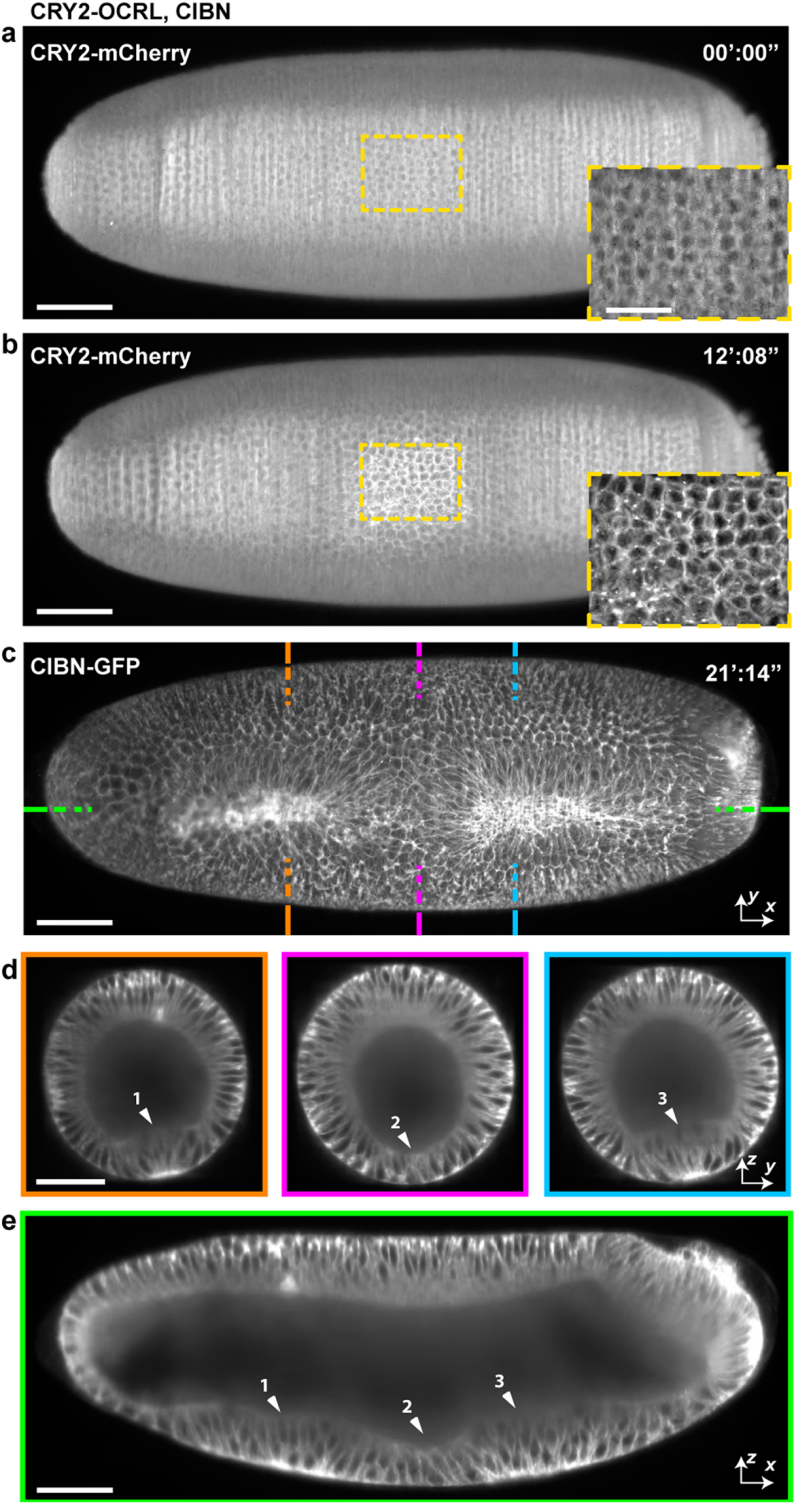
4D Imaging of localized optogenetic inhibition of formation of the ventral furrow. (**a**) Ventral view of a *Drosophila* embryo before and **(b)** after 9 cycles of photo-activation. Anterior is on the left and ventral is *en face*. Insets show the activated region localized in the mid-ventral side of the embryo. CRY2-dOCRL activation results in the recruitment and an increased mCherry signal at the cell cortex. **(c)** Maximum intensity projection of the embryo during ventral furrow formation. Dashed color coded lines refer to transversal **(d)** and sagittal **(e)** sections. Arrowheads indicate non-activated (1,3) and activated (2) regions. Scale bars 50 μm.

During *Drosophila* gastrulation, contractility is established on the ventral side of the embryo, driving cell apical constriction and triggering ventral tissue folding. If PI(4,5)P_2_ is depleted from the cortex of ventral cells before gastrulation, apical constriction is inhibited and ventral tissue folding compromised^13^. To confirm these results, we mounted the embryo on MuVi-SPIM and exposed a rectangular region on the ventral side of the embryo to IR fs light (see dashed box in Fig. 5a). After photoactivation (5 activation cycles of 1 min per cycle on average) CRY2-dOCRL was recruited at the cell cortex and depleted from the cytoplasm (Fig. 5b and Supplementary Movie 6). At the onset of gastrulation, photoactivated cells failed to constrict apically (Fig. 5c). *In toto* imaging (see Fig. 5d,e and Supplementary Movie 7) showed that anterior and posterior cells constricted and formed a furrow (Fig. 5d, left and right panels), whereas photo-activated cells failed to constrict and to elongate along the apical-basal axis (Fig. 5d, central panel).

**Figure 6:**
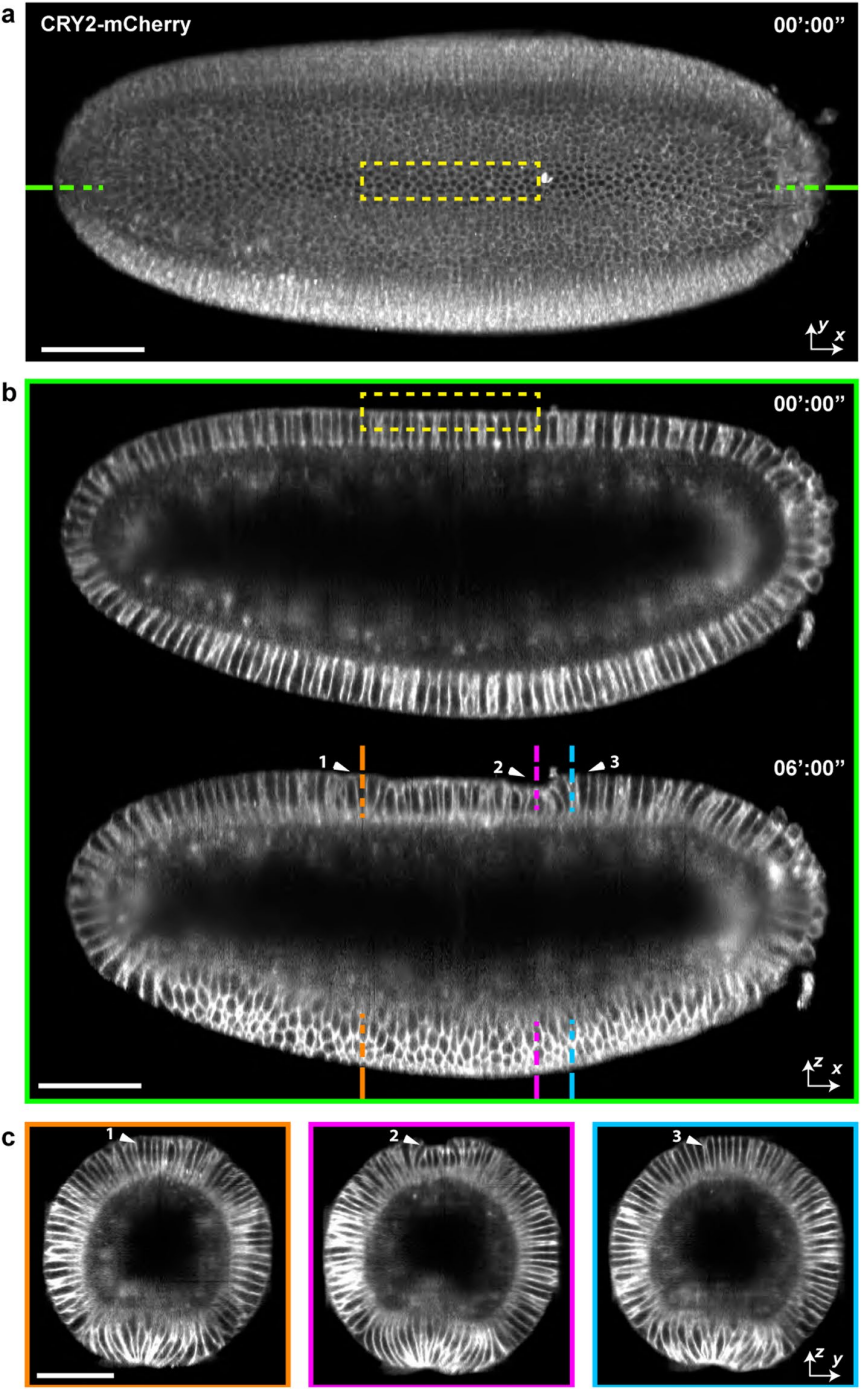
4D Imaging of localized optogenetic promotion of dorsal apical contraction. (**a**) Dorsal view of a *Drosophila* embryo before onset of activation. Yellow dashed box depicts the region of activation. **(b)** Sagittal section of the same embryo as marked by dashed green lines in (**a**), showing the embryo before and 6 minutes after # cycles of photo-activation. Anterior is on the left and ventral is facing down. **(c)** Maximum intensity projection of the embryo during ventral furrow formation, with regions color-coded according to dahed lines in lower panel of (**b**). Arrowheads indicate non-activated (1,3) and activated (2) regions. Scale bars 50 μm.

We then promoted cell constriction and cell apical-basal shortening by selectively inducing the activation of the Rho pathway. In this case, the CRY2-CIB1 system was used to recruit RhoGEF2-CRY2 to the plasma membrane^12^. After targeting a group of cells located at the dorsal side of the *Drosophila* embryo, the dorsal tissue furrowed (Fig. 6b–d and Supplementary Movie 9). We then imaged and monitored cell displacement over the full embryo. The dorsal synthetic furrow disappeared after 30 min of embryo gastrulation (Supplementary Movie 9). In conclusion, pulsed IR laser coupled to MuVi-SPIM is an effective tool to selectively activate photo sensitive proteins in 4D. Temporal, spatial and protein specific activation can induce morphogenetic perturbation or trigger synthetic tissue shape changes that can be monitored at the embryo scale with cellular resolution.

## Discussion

Laser-based manipulation coupled to live imaging is a powerful tool to perturb and monitor living samples. While IR fs laser coupled to confocal imaging units allow to manipulate and visualize only a limited region of a sample, we present a new system based on a IR fs laser coupled to MuVi-SPIM that allows to target any region of an embryo with subcellular resolution and to perform *in toto* live imaging at high spatial and temporal resolution for long periods of time. The system is controlled by a graphical user interface (GUI) by which the user can define a target ROI and alternating cycles of laser manipulation and 4D imaging.

We have shown that with this system, while imaging with MuVi-SPIM, we can (1) ablate structures in the embryo, (2) perform high spatially resolved dissection to selectively target cellular sub-micrometer structures (3) generate fixed barriers to impede global cell movements and tissue flows during embryo development, (4) perform two-photon optogenetics to modulate protein distribution and perturb tissue morphogenesis while performing *in toto* imaging.

In line with recent innovations to shape the light-sheet^47,48^ and correct image aberrations^49^, an interesting future development of our integrated framework is to combine these innovations with laser manipulation to better resolve and target structures in the 3D space or in time.

Since biological samples are generally complex 3D structures, it will be important to further develop automatic 3D sample shape recognition^50,51^ and rapid 2D/3D ROI definition over complex 3D structures while imaging. This will allow in the future to precisely target regions of interest that extend over curved surfaces and volumes in the embryo.

## Methods

### Software

To perform ablation experiments, laser power, exposure time and beam position on the specimen must be finely controlled in an automated fashion. To dynamically modulate the laser power, we use an attenuator unit comprised of a λ/2 plate mounted on a rotation stage followed by a fixed Glan-Thompson Prism (GLTP). Serial communication through RS232 commands controls the rotation stage with the λ/2 plate resulting in a controlled laser power modulation after the GLTP. A digital signal sent to a shutter controls the laser exposure time. To dynamically control the position of the laser beam on the specimen, a two galvanometric mirror set is positioned in such a way that the conjugated plane of the back focal plane of the detection objective lies between both mirrors. This way it is possible to steer the focused beam on the object plane which in MuVi-SPIM matches the imaging and light-sheet illumination planes. We can then access any region of a sample since this is mounted on top of automated stages which allow translation and rotation. The λ/2 rotation stage the shutter and the two galvanometric mirrors are controlled via a custom-written LabView interface.

The software allows us to define the experiments as a series of imaging events. Each event consists of a combination of channels and stacks, as well as the timing information. The channels define the state of all devices (e.g. lasers, filers), while the stack provides the positional information. The latter consists of 4 starting coordinates (x, y, z_start_, r), an end coordinate (z_end_), and the step size. The stage will move from z_start_ to z_end_ in increments of the step size between two exposures of the camera.

For each channel it is possible to optionally define a photomanipulation region by drawing on the live images. The calibration procedure that allows this interaction is described in Supplementary Note 3. During the acquisition the photomanipulation module will perform the 2D scanning of the desired pattern while the stack is acquired as described above. This allows us to target single points, 2D surfaces (when z_start_ = z_end_), and 3D regions (when z_start_ ≠ z_end_). By combining multiple imaging events with the appropriate timing information, it is possible to set up an experiment with a single or multiple photomanipulation events followed by or interleaved with regular time-lapse imaging.

### *Drosophila* embryo preparation and mounting

The following crosses were obtained by standard fly husbandry methods. All stocks and cages were maintained at 22 °C. Flies of the desired genotype were collected in cages with apple juice plates and yeast paste. For embryo collection, the embryos were identified and mounted using a standard stereomicroscope under transmitted illumination. For light sensitive lines, cages were kept and manipulated in the dark. Additionally, the microscope light source was replaced with a conventional red-emitting LED lamp to prevent unwanted photo-activation. Following selection under halocarbon oil, the embryos were dechorionated with 90% sodium hypochlorite for 2 minutes, washed with water and mounted in PBS. Mounting procedure was followed according to previous work^46^. To show the deep nuclear ablation and the cauterization experiments, the following lines were used: NUP107-mRFP and His2Av-GFP (Bloomington stock number 35518). To show the effect of dOCRL plasma membrane recruitment on the ventral epithelium upon photo-activation: w[*]; P[w+, UASp > CIBN::pmGFP]/+; P[w+, UASp > mCherry-CRY2::dOCRL]/ P[w+, Osk > Gal4::VP16]. To show the effect of RhoGEF2 plasma membrane recruitment on the dorsal epithelium upon photo-activation: P[w+, sqhp > Gap43::mCherry]/+; P[w+, UASp > CIBN::pmGFP]/+; P[w+, UASp > RhoGEF2(DHPH)-CRY2::mCherry]/ P[w+, Osk > Gal4::VP16].

### Fly stocks

w[*]; P[w+, UASp > CIBN::pmGFP]/Cyo; Sb/TM3, Ser. Membrane-anchored CIBN additionally fused to EGFP[13]. w[*]; P[w+, IF/Cyo; P[w+, UASp > mCherry-CRY2::dOCRL]/TM3, Ser. Light sensitive Drosophila dOCRL together with CRY2 additionally fused to mCherry[13]. w[*]; If/CyO; P[w+, Oskp > Gal4::VP16]/TM3, Ser. Maternally deposited Gal4 protein driven by the Oskar promoter (kindly provided by Imre Gaspar, stock 44242 Bloomington). w[*];IF/CyO; P[w+, UASp > RhoGEF2-CRY2::mCherry]/TM3, Ser. Light sensitive Drosophila RhoGEF2 (only PHDH domains) together with CRY2 additionally fused to mCherry13^12^. P[w+, sqhp > Gap43::mCherry]/FM7; GAP43 membrane marker tagged with mCherry and driven only by the sqh promoter^12^.

### Zebrafish embryo lines and preparation

The following transgenic lines were used: Synaptophysin-sfGFP to label neurons Tg(NBT::ΔLexPR-LexOP::Syp-sfGFP) and Tg(pU.1::GAL4-UAS::TagRFP) for microglia^52^. The Tg(NBT::ΔLexPR-LexOP::Syp-sfGFP) line, was generated by cloning the neuronal beta tubulin promoter (NBT) in front of the ΔLexPR-LexOP system^52^ followed by the synaptic vesicle synaptophysin fused to super-folder GFP. Imaged embryos were 5 days post fertilization (dpf). Illumination laser powers were chosen such that further increase would only lead to stronger background signal, leading to 160 μW on each illumination path (measured at the back aperture of the illumination objectives).

### Live imaging and perturbations

All fluorescence imaging was performed with MuVi-SPIM^2^. For the experiments of cauterization, stacks were taken every 15 seconds. During deep nuclear ablation, two dual illumination and dual detection stacks at 0 and 90 degrees sample rotation were acquired every 90 seconds. Exposure time for each plane was 50 ms. In order to minimize phototoxicity and increase imaging speeds, images were taken at 3 μm steps.

### Laser manipulation parameters

Ablation parameters changed from experiment to experiment. For cauterization, the average infrared laser power used was kept around 400 mW – yielding a power density of around 1.9 ⋅ 10^12^ W/cm^2^ at the focus - and exposure times of 40 ms. Cumulative thermal effect of fs pulses can lead to temperature increase and the corresponding stitching of the different tissues. All regions of ablation consisted of pre-defined lines which were scanned automatically by the 2D steering galvanometric mirror system. After image acquisition was completed, the capillaries containing the embryos were removed from the microscope, the embryos removed from the embedding gel and prepared for electron microscopy through high pressure freezing and freeze substitution. EM imaging was performed at the electron microscopy facility at EMBL Heidelberg. During deep nuclear ablation, nuclei were ablated by exposing each target 4 times on average with 800 mW, each point ablation with an exposure of around 15 ms. Zebrafish soma ablation was performed with 880 mW IR average laser power and 5 times 9 ms exposure time, this being enough to generate a micro-cavitation bubble inside the neuronal cell body, which disappears shortly afterwards. For axon ablation we measured 604 mW IR average laser power at the back aperture of the detection objective and 3 ms exposure time. For the optogenetics experiments a 780 nm pulsed laser (Femto Fiber ultra 780 TOPTICA Photonics AG) working at around 150 fs pulse width and 80 MHz repetition rate was used for performing the activation of the CRY2-CIBN system: For the optogenetic experiments we used the 780 nm laser wavelength to increase the absorption rates as previously reported^32,33^. The laser was kindly provided by Toptica Photonics AG. In all experiments, scanning of the region was repeated several times – typically every 30 s to 1 min during 10 min maximum – until the onset of ventral furrow was observed. After that, *in toto* imaging was initiated, while maintaining activation at more sparse timings.

### Electron microscopy

For EM analysis, the embryos were high-pressure frozen (HPM010 AbraFluid), using 20% dextran as cryoprotectant. The embryos were pierced with a needle in a cryo-microtome chamber (Leica EM FC6) at −160 °C to facilitate freeze substitution^53^. Embryos were then freeze-substituted (EM-AFS2 - Leica Microsystems) with 0.3% Uranyl Acetate (UA), 0.3% Glutaraldehyde and 3% water in acetone at −90 °C for 48 h. The temperature was then raised to −45 °C at 2 °C/h and samples were further incubated for 16 h. After rinsing in acetone, the samples were infiltrated in Lowicryl HM20 resin, while raising the temperature to −25 °C and left to polymerize under UV light for 48 hours at −25 °C and for further 9 hours while the temperature was gradually raised to 20 °C(5 °C/h). 70 nm cross sections were cut from the polymerized resin block in the region of the embryo cauterization and picked up on formvar coated slot grids. Tiled 2D images were acquired with a FEI Tecnai F30 electron microscope. Images were then stitched using Etomo (Imod software package^54^).

## Supplementary information

Video 1

Video 2

Video 3

Video 4

Video 5

Video 6

Video 7

Video 8

Video 9

Supplementary information
