# Supplementary material for: Cell and tissue manipulation with ultrashort infrared laser pulses in light-sheet microscopy
Source: Sci Rep. 2020 Feb 6;10:1942. doi: 10.1038/s41598-019-54349-x (PMC7005178; doi:10.1038/s41598-019-54349-x)
Supplement: Supplementary file 10 — Supplementary information [file 41598_2019_54349_MOESM10_ESM.pdf]

Cell and tissue manipulation with ultrashort infrared laser pulses in light-sheet microscopy

Gustavo de Medeiros[1,*], Matteo Rauzi [1,2,*,§], Dimitri Kromm[1,3], Balint Balazs[1,4], Nils Norlin[1], Stefan Günther[1], Emiliano Izquierdo[1], Paolo Ronchi[1], Shinya Komoto[1], Uros Krzic[1], Yannick Schwab[1], Francesca Peri[1,5], Stefano de Renzis[1], Maria Leptin[1] & Lars Hufnagel[1, §]

[1] European Molecular Biology Laboratory Heidelberg, Meyerhofstrasse 1, 69117 Heidelberg, Germany.
[2] Université Côte d'Azur, CNRS, INSERM, iBV, France.
[3] Collaboration for joint PhD degree between EMBL and Heidelberg University, Faculty of Biosciences
[4] Present address: Luxendo GmbH, Kurfürsten-Anlage 58, 69115 Heidelberg
[5] Present address: Institute of Molecular Life Sciences, University of Zurich, Winterthurerstrasse 190, CH-8057 Zurich, Switzerland

* These authors contributed equally.
§ Corresponding authors: M.R. (email: matteo.rauzi@univ-cotedazur.fr) and L.H. (email: lars.hufnagel@embl.de)

Supplementary information

Note1 - Optical setup for ablation and cauterization experiments

The infrared laser source (Mikan laser from Amplitude Systèmes) denoted **MPL** in the Fig 1 has its main properties shown in **Supplementary Table 1**. Pulse trains pass through a λ/2 waveplate with special coating for 1030 nm and a Glan Thompson prism (GLTP) (WPH05M-1030 and GL10-B, both from Thorlabs), the former being attached to a motorized rotation stage (M116-DG stage with a C863-DC Mercury controller, from Physik Instrumente). The λ/2 plate rotates the pulses' polarization, and the Glan Thompson prism allows polarization components with one particular orientation to pass through as the extraordinary beam, all other components being redirected outside the optical path. A mechanical shutter (**S**, Uniblitz VS25 25 mm

shutter and a VMMD-1 shutter driver, from Vincent Associates) is placed in front of the pulsed laser (Mikan, Amplitude Systèmes), at an angle so that when closed the reflected infrared beam can be directed into a beam dump (not shown). Together, these three parts control exposure time and the laser power used. All mirrors (**M**) are broadband dielectric (Thorlabs BB1-E03, coated for 750 - 1100 nm spectral range). Infrared laser pulses are then expanded spatially once, by a factor of almost 2, with two (**L1**,**L2**) infrared-coated lenses (focal lengths 30 mm and 50 mm. LB1757-B and LB1471-B, Thorlabs), and enter a specially designed cube which houses the 2D steering optics. The system is composed of a right angle dielectric mirror (MRA10E03, Thorlabs) which guides the infrared pulses to a 2D galvanometric mirror set (silver coated) from Cambridge Technologies (comprised of two VM500+ motors with a 673 dual axis servo controller), the responsible mirrors for steering (translating) the focal spot of the focused beam in the FOV of the sample (**y-GSM**, **x-GSM**). Although the mirrors do not have special coating for the wavelengths utilized, unchanged pulse lengths were measured before and after the system – more specifically, just before the first beam expansion lens and after the dichroic mirror (**L_1** and **DM** in **Figure 1**, respectively). Pulse length measurements were performed with a PulseScout autocorrelator from Spectra Physics. Furthermore, the aforementioned cube also houses the first lens of the second beam expansion (**L3**), and the whole cube mounted on optical rails (SYS65, Owis). This promotes the flexibility for axial alignment of the focal spot, which can be important when a medium with different refractive index needs to be used in the sample chamber in order to ensure *in vivo* imaging. The housing is depicted in **Supplementary Figure 1a**. The second beam expansion (**L3**, **L4**; roughly 8 times expansion with 25.4 mm LB1761-B from Thorlabs and a 200 mm NIR coated 49-506 from Edmund Optics) also acts as a

relay system, imaging the back focal plane of the objective to a plane located in between the two galvanometric mirrors. This way each mirrors' angular movement ensures proper translation of the focal spot on the field of view. The fully expanded beam with a diameter of roughly 13.4 mm (at $1/e^2$) is directed to one of the detection objectives of our multiview light-sheet setup (**DO_1**; 25X/1.1 numerical aperture (NA), water immersion, infrared corrected, CFI Apo LWD, Nikon) via a 5 mm thick dichroic filter (**DM**; AHF Analysentechnik F73-877). Due to the geometrical configuration of the four objectives in MuVi-SPIM, both detection objectives share an overlapping focal plane. This can be particularly dangerous when focusing high intensity pulsed laser with one of the objectives, as due to symmetry the infrared pulses are going to be focused on the opposing camera through the second objective. It is therefore crucial that the opposing detection path is protected from transmission of infrared wavelengths. The same caution is also necessary for the detection path where the infrared laser is coupled to: also this camera has to be protected from any back reflections. For these reasons, extra 680 nm short-pass (SP) filters from Semrock (depicted as **$IRF_{1,2}$**; FF01-680/SP-25) have been added, one to each detection path, which blocks the pulsed infrared light with an optical density of 10 (OD10). The dichroic mirror and the short pass filter are mounted in a dedicated turret with easy access, so that the microscope can be also used in the classical 1p configuration without ablation, and each optical element can be mounted separately. From this configuration, and given that the radius of the laser beam at the focal point of the objective (based on the Rayleigh criterion)[1]:

$$r = \frac{0.61 \cdot \lambda}{NA} = \frac{0.61 \cdot 1025}{1.1} \cong 568\ \mathrm{nm}$$

where NA is the numerical aperture of the objective and λ the laser wavelength, we can calculate the maximal energy density $\hat{E}_{pulse}^{max}$ at the focus (considering 50% of light losses):

$$\hat{E}_{pulse}^{max} = 0.5 \cdot \frac{E_{pulse}}{\pi r^2} \cong 0.5 \cdot \frac{20.3 \text{ nJ}}{1.0 \cdot 10^{-8} \text{ cm}^2} = 1.0 \text{ J} \cdot \text{cm}^{-2}$$

Finally the peak power density $\hat{P}_{pulse}$can be calculated by taking into account the temporal width of the pulse, τ_{pulse}:

$$\hat{P}_{pulse} \cong \frac{\hat{E}_{pulse}^{max}}{\tau_{pulse}} = \frac{1.0 \text{ J} \cdot \text{cm}^{-2}}{180 \cdot 10^{-15} \text{ s}} = 5.5 \cdot 10^{12} \text{ W} \cdot \text{cm}^{-2}.$$

SupplementaryTable1 – Properties of the pulsed laser sources

	Mikan	FF ultra 780	
wavelength λ	1025	780	nm
pulse duration τ_{pulse}	200	140	fs
average power $<P>$	1.1	0.5	W
repetition rate f_{rep}	54	80	MHz
pulse energy E_{pulse}	20.3	6.2	nJ

Note 2 - Optical setup during optogenetic experiments

For the optogenetic experiments presented in **Figure 5**, certain changes on the optics utilized for activation were performed, since the CRY2 optogenetic system has very low absorption for two-photon processes with a 1025 nm pulsed laser[32]. Therefore, we utilized a 780 nm pulsed laser from Toptica Photonics (FemtoFiber

ultra 780), which delivered ~140 fs pulses with 80 MHz repetition rate. Due to the change in wavelength, a different λ/2 plate needed to be used for more effective laser power control (Thorlabs WPH05M-780). In addition, we used lower magnification detection objectives (Olympus XLUMPLFLN 20XW) during the optogenetic experiments.

Note 3 – Calibrating the XY-galvanometric system

Alignment of the XY-galvanometric system and the position of the focus on the image plane is performed by mapping the pixel coordinates of the camera image to voltages on the galvanometric mirrors that would place the image of the fluorescent spot to the correct position. This greatly simplifies the definition of the manipulation regions, as it allows the users to directly draw the regions of the live images.

The deflection angle of the beam linearly depends on the voltage applied to the galvanometer. Due to the small scanning angles, we can approximate tan $\varphi \approx \varphi$. Thus, a linear transformation can be used to describe the relationship between pixel coordinates (**p**) and voltage coordinates (**u**):

$$\mathbf{u} = \mathbf{A}\boldsymbol{p},$$

$$\begin{bmatrix} u_x \\ u_y \\ 1 \end{bmatrix} = \begin{bmatrix} a_{xx} & a_{xy} & t_x \\ a_{yx} & a_{yy} & t_y \\ 0 & 0 & 1 \end{bmatrix} \begin{bmatrix} p_x \\ p_y \\ 1 \end{bmatrix},$$

where u_i represent the voltages to be applied to the galvanometers, p_i are the pixel coordinates, a_{ij} are the 2D affine transformation parameters, and t_i are the translation components.

During the calibration procedure the transformation matrix $\boldsymbol{A}$ is determined by measuring the 2-photon spot coordinates at different galvanometer settings, and performing a 2D linear regression. During normal operation the users can specify the laser position and ROIs in pixel coordinates which will be automatically transformed to voltage coordinates and sent to the galvanometric scanners.

The calculations follow similar procedures as shown in previous work[4], by extending it to 2D. To summarize the procedure step by step:

1. Remove the sample from the chamber and fill the chamber with fluorescent medium (0.001% methylene blue solution).
2. Set both galvanometers to 0 V, and make sure that the fluorescent spot is visible when the laser is on, and it is in the middle of the field of view.
    a. If the spot is not in the middle, either rotate the scanning mirrors to the appropriate position, or apply a small offset voltage. In either case, (0, 0) should correspond to the center of the image.
3. Record a set of 20 images for 5 different scanner positions, *e.g.* (0 V, 0 V), (0 V, 0.5 V), (0 V, -0.5 V), (0.5 V, 0 V), (-0.5 V, 0 V)
4. For each position average the acquired images to increase the signal-to-noise ratio.
5. For each position, perform a 2D Gaussian fit, to determine the center of the spot.
6. Perform 2D linear least square fitting on the preset voltage coordinates ($\boldsymbol{u_i}$) and the recorded pixel coordinates ($\boldsymbol{p_i}$) to obtain the transformation parameters **A**.

A LabView implementation of steps 4–6 of the above procedure is provided in the **Supplementary Code** as a VI snippet. It can be viewed as a simple image file, or directly loaded into LabView and executed. For more information see: http://www.ni.com/tutorial/9330/en/. In order to run the example code, it is necessary to install the h5labview2 library, which can be obtained from the following website: http://h5labview.sourceforge.net/.

Supplementary Figure Caption

Supplementary Figure 1: **LabView diagram presenting the calibration code.** LabView VI snippet of the calibration software. It can be viewed as a simple image file, or directly loaded into LabView and executed. For more information see: http://www.ni.com/tutorial/9330/en/. In order to run the example code, it is necessary to install the h5labview2 library, which can be obtained from the following website: http://h5labview.sourceforge.net/.

Movie captions

Supplementary Movie 1: Tracking of ablated nuclei. 4 cross sections of the same embryo marked with NUP-RFP as shown in **Figure 2a.** Each of the ablated nuclei (numerated accordingly) is followed throughout the entire acquisition (as presented in **Figure 2c).** All ablated nuclei end up in the middle of the yolk sac. Scale bar 50 µm.

Supplementary Movie 2: Ablation of a neuronal body inside the brain of a zebrafish embryo (pu.1-mCherry and Synaptophysin-sfGFP markers). Time 00:00 corresponds to the first stack acquired right after laser ablation was performed. A single microglia is then directed to the site of injury engulfing the ablated neuron. Scale bar 10 µm.

Supplementary Movie 3: Ablation of a neuronal body inside the brain of a zebrafish embryo (pu.1-mCherry and Synaptophysin-sfGFP markers). Local damage of an axon leads a nearby microglia to the site of injury. After almost 15 minutes four microglia are on the site of injury. Scale bar 20 µm.

Supplementary Movie 4: Laser cauterization at the surface and inside the embryo. Cylindrical projection of a *Drosophila melanogaster* embryo expressing GAP43-mCherry. Ventral is in the middle and dorsal is on the top and bottom. On the left and right the *Drosophila* embryo layers are shown at 4 µm and 12 µm distance respectively from the vitelline membrane: this is to best depict the depth at which each cauterization (bright lines on each lateral side of the embryo) was performed. Only the cauterization close to the vitelline membrane forms a fix boundary impeding cell displacement. Scale bar 100 µm.

Supplementary Movie 5: Cross section of the embryo shown in **Supplementary Movie 4**. The mesoderm shifts towards the fixed boundary. The shift is a consequence of the differential left/right ectoderm displacement. Scale bar 50 µm.

Supplementary Movie 6: Single-plane timelapse showing the activation of CRY-dOCRL that is eventually recruited at the cell cortex (**Fig. 5**). At the end of the movie

ventral furrow forms at the top and bottom but not in the activated region. Scale bar 50 µm.

Supplementary Movie 7: 3D view of the *Drosophila* embryo in the activation CRY-dOCRL experiment shown in **Fig. 5**. Scale bar 100 µm.

Supplementary Movie 8: Cylindrical project and cell tracking of the *Drosophila* embryo in the activation CRY-dOCRL experiment shown in **Fig. 5**. Maximum cell speed is 0.12 µm/s. Scale bar 100 µm.

Supplementary Movie 9: Cylindrical project and cell tracking of the *Drosophila* embryo in the activation CRY-RhoGEF2 experiment shown in **Fig. 6**. Lines are cell tracks and color is cell speed. Maximum cell speed is 0.22 µm/s. Scale bar 100 µm.

1 Rayleigh, L. Wave theory of light. *Sci. Pap. John William Strutt Baron Rayleigh* **3**, 47–189 (1902).

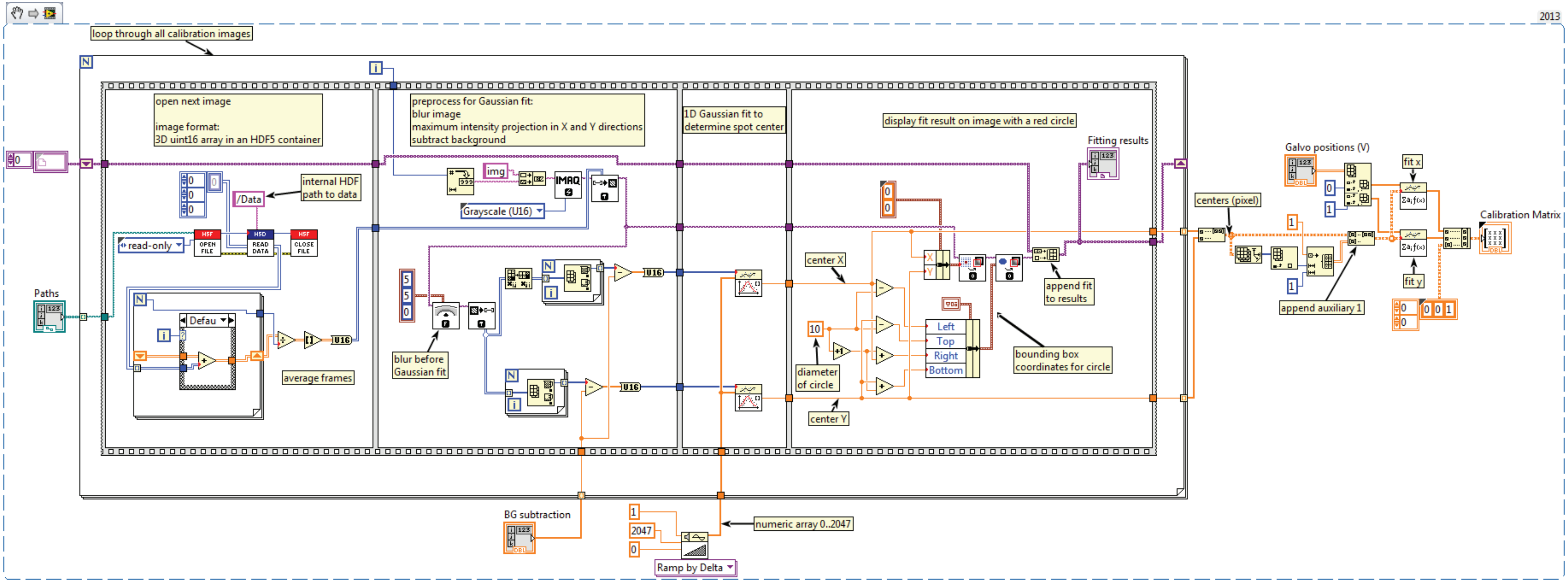

Supplementary Figure 1
